# Modified technique of Hepatojejunostomy for biliary tract reconstruction after resection of tumors affecting the perihilar region: a case series

**DOI:** 10.1186/s12893-024-02393-4

**Published:** 2024-04-10

**Authors:** Yi Kuang, Ran Ji, Tao Yuan, Menggang Liu

**Affiliations:** 1 Department of Hepatobiliary Surgery, Daping Hospital, Army Medical University, Chongqing, 400042 China; 2 Department of Burn and Plastic Surgery, The First Affiliated Hospital of Chongqing Medical University, Chongqing, 400042 China; 3 Department of Hepatobiliary and Pancreatic Surgery, The People’s Hospital of Chongqing Liang Jiang New Area, No. 199, Renxing Road, Chongqing, Liangjiang New District 400042 China

**Keywords:** Perihilar cholangiocarcinoma, Biliary tract reconstruction, Hepatojejunostomy, Perihilar tumors, Postoperative complications

## Abstract

**Backgrounds:**

Radical resection is the most effective treatment for perihilar tumors. Biliary tract reconstruction after resection is one of the key steps in this surgery. Mucosa-to-mucosa cholangiojejunostomy is traditionally performed, in which the bile ducts at the resection margin are separately anastomosed to the jejunum. However, this approach is associated with long operative time and high risk of postoperative complications. The present study presents a modified technique of hepatojejunostomy and its outcomes.

**Methods:**

The data of patients who underwent hepatojejunostomy using the modified technique at the Department of Hepatobiliary Surgery, Daping Hospital, Army Medical University, Chongqing, China, from January 2016 to December 2021, were retrospectively analyzed.

**Results:**

A total of 13 patients with perihilar tumors underwent R0 resection and bilioenteric reconstruction using the modified hepatojejunostomy technique during the study period. During the operation, the alignment of the bile duct stumps was improved, the posterior wall of the anastomosis was reinforced, internal stents were placed in the smaller bile ducts, external stents were placed in the larger bile ducts, and hepatojejunostomy was performed using 4 − 0 prolene. No serious postoperative complications, such as death or bile leakage, occurred during the hospitalization. Furthermore, there were no cases of biliary stricture or cholangitis after the six-month follow-up period.

**Conclusion:**

The modified hepatojejunostomy technique is a safe and effective technique of biliary reconstruction after the resection of perihilar tumors. This can be easily performed for difficult cases with multiple bile ducts that require reconstruction after resection.

## Introduction

Bilioenteric reconstruction is one of the key steps of surgery for perihilar tumors and benign perihilar diseases. The extent of the intrahepatic bile duct involvement has been an important role when deciding on the implementation of radical surgery for perihilar cholangiocarcinoma (pCCA) [1]. A number of studies have defined bilateral tumor invasion into the secondary or tertiary intrahepatic biliary radicles as an unresectable disease [2–4].

With the advancement of surgical techniques, the local recurrence of pCCAs has significantly decreased, and the overall survival has considerably improved. However, due to extended hepatic resections for complex pCCAs, a number of secondary or tertiary biliary radicles require reconstruction. Traditional cholangiojejunostomy is associated a high incidence of postoperative complications, especially anastomotic leaks and stenosis, in pCCAs [5, 6]. Cholangiojejunostomy is traditionally performed by end-to-side anastomosis one by one, or anastomosis of the main branch, accompanied by ligation of the smaller branches. To overcome the drawbacks of traditional cholangiojejunostomy, various modified techniques have been developed in the past decades. Several types of biliary reconstruction techniques have been reported, such as basin-like Roux-en-Y cholangiojejunostomy [7], multiple Roux-en-Y hepaticojejunostomy [8], cluster hepaticojejunostomy [9, 10]. Kasai portoenterostomy, which was proposed for the treatment of congenital biliary atresia, was also applied in pCCA surgery [11–13]. A number of scholars have also made some improvements based on the Kasai operation [14, 15].

To reduce the difficulty of the operation and incidence of postoperative anastomotic complications, the bilioenteric anastomosis technique was modified after taking into consideration the advantages and disadvantages of various types of reported hepatojejunostomy, and this was applied after the resection of pCCAs and other types of tumors affecting the perihilar region.

## Materials and methods

### Patients

The data of patients who underwent the modified hepatojejunostomy at the Department of Hepatobiliary Surgery, Daping Hospital, Army Medical University, Chongqing, China, from January 2016 to December 2021, were retrospectively analyzed. The indications of the modified hepatojejunostomy were as following: (1) resection of tumors located at or near the hepatic hilum; (2) the number of bile duct stumps ≧ 3 and/or the diameter of the ducts ≦ 3 mm; (3) the bile duct stumps cannot be reconstructed into one duct. Patients who were < 18 years old, had incomplete data, or were lost to follow up were excluded. A written informed consent was obtained from each patient before the surgery. The present study was approved by the Ethics Committee of Daping Hospital (IRB number: 292).

### Surgical procedure

**Fig. 1:**
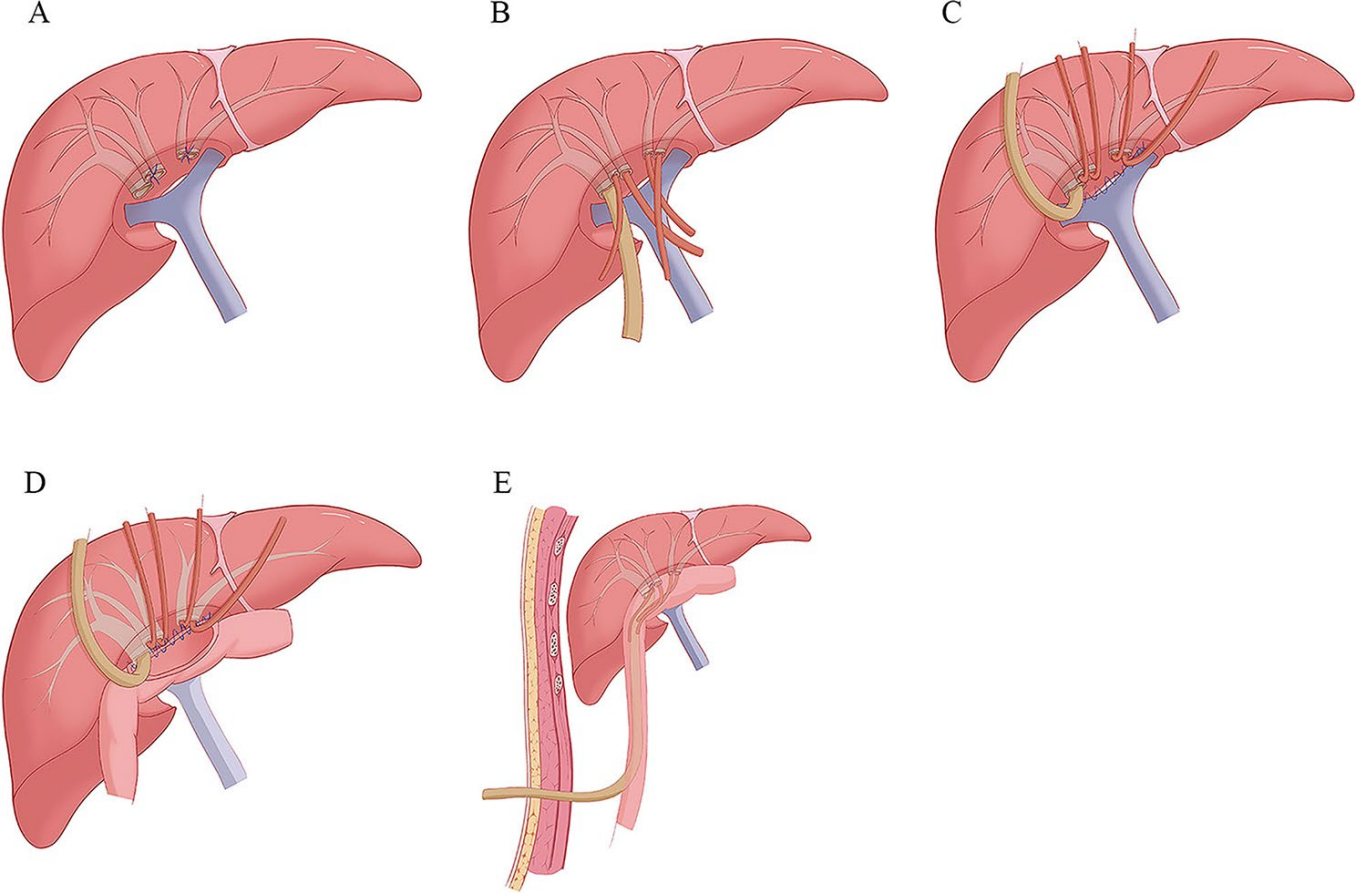
Key steps for the modified hepatojejunostomy

**Fig. 2:**
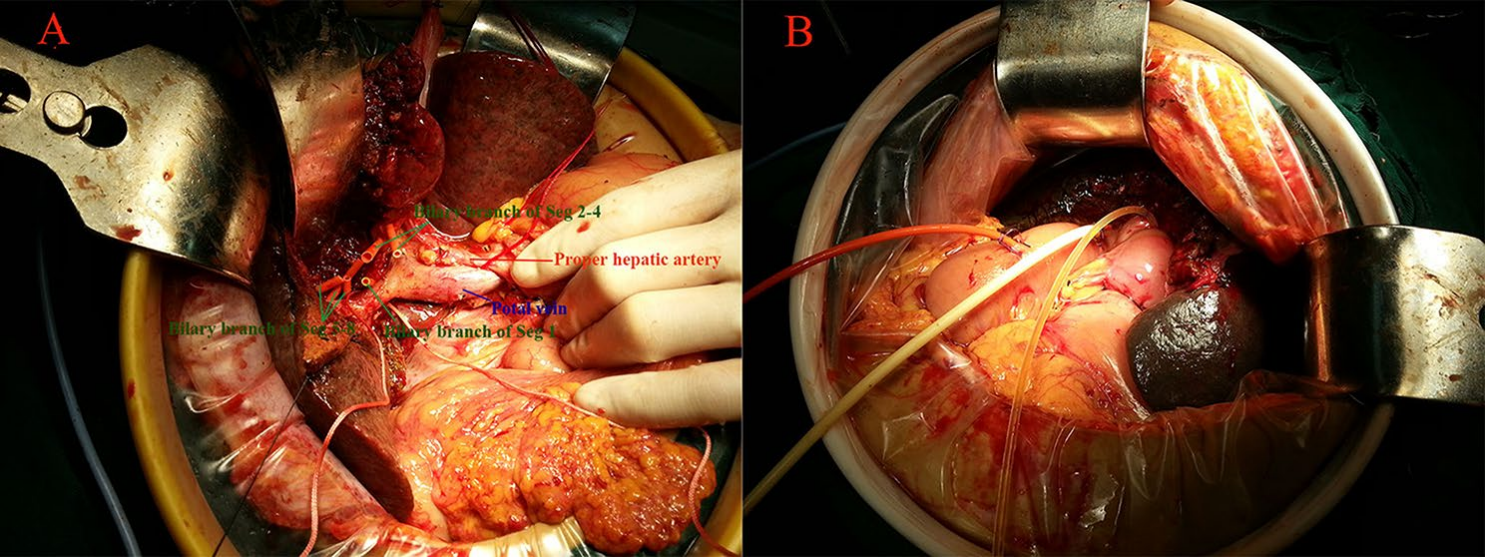
Intraoperative images show the multiple biliary stumps after resection (**A**), and the bilioenteric anastomosis (**B**). The right hepatic artery, invaded by tumor, was ligated but the right lobe was preserved in this case. In order to ensure the blood supply of the right lobe, the ligaments of the right side

A reverse “L”-shaped incision was made on the right upper quadrant, and the abdominal cavity was examined to search for any distant metastasis, and determine the possibility of resection. Then, radical tumor resection and lymph node dissection were routinely performed. Afterwards, the left and right branches of the portal vein and hepatic artery were skeletalized, and the invaded vessels were resected and reconstructed, depending on the extent of the tumor infiltration. The extent of liver resection was determined according to the tumor distribution, liver status, and general condition of the patient. Intraoperative frozen section of the proximal bile duct margin was performed to confirm the R0 resection. Hepatojejunostomy was performed according to the following steps, after confirming the hemostasis:

1. The biliary stumps were clearly identified and exposed. If conditions permitted, the posterior wall of the biliary stumps located at the lower side of the section was aligned in a line as much as possible (Fig. 1A). According to the diameter of the bile ducts, suitable silicone or latex tubes were used as stents, and these were fixed to the wall of the bile ducts using absorbable sutures (Figs. 1B and 2).

2. In order to eliminate the gap between the branches of the portal vein and the cut surface of the liver, the walls of the branches of the portal vein were sutured to the adjacent liver tissue before anastomosis (Fig. 1C).
3. The Roux-en-Y jejunal loop was constructed and lifted to the porta hepatis through the mesocolon. An incision of appropriate size that corresponded to the total area occupied by all bile duct stumps was made on the Roux-en-Y jejunal limb for the anastomosis.
4. The full thickness of the posterior wall of the jejunum was sutured to the posterior wall of the lower bile ducts, the adjacent connective tissue, the adjacent liver tissue, and the wall of the portal vein using 4 − 0 prolene (Fig. 1D).
5. The full thickness of the anterior wall of the jejunum was continuously sutured to the anterior wall of the upper bile ducts and the adjacent liver tissue using 4 − 0 prolene (Fig. 1E).
6. Silicon or latex tubes were used as the external biliary stents according to its diameter. The small and thin bile ducts were supported by thin silicone tubes with a length of 8–10 cm, and the extrahepatic part of the stents was placed in the jejunum cavity. For larger and thicker bile ducts, a silicone or latex tube was placed in the bile duct. Then, a side hole was opened in the extrahepatic part of the stent tube, and was placed in the thicker or main bile duct. Then, the distal end of the stent was drawn out, at 10 cm from the anastomosis, and fixed to the intestinal wall (Figs. 1E and 2B).

All bile duct stumps were not sutured together. If the bile duct stump was thin or of poor quality, this was left alone within the wide hepatojejunostomy, and was not forcibly sutured to the jejunum. After the anastomosis, the abdominal cavity was washed, and the anastomosis was checked for bleeding and bile leakage. Drainage tubes were placed below and above the anastomosis, when necessary.

### Postoperative care

All patients were postoperatively treated with prophylactic antibiotics, hepatoprotection drugs, and supportive care, to maintain water and electrolyte balance. At one day after the operation, the nasogastric tube was removed, and the patient was allowed to drink water. After the recovery of intestinal peristalsis, the patient was started on liquid diet. The abdominal drain was removed early when there was no or minimal drainage output. If complications occurred, treatment was given based on the type of complication.

Discharge criteria: sufficient dietary intake to meet physiological needs, no special medical treatment was needed, and all postoperative complications were treated.

### Follow up after discharge

The diagnosis of biliary leak was based on the clinical symptoms, the content and color of drain fluid. The external biliary tube was removed at 4–6 weeks after surgery. Abdominal computed tomography (CT) or magnetic resonance imaging (MRI) was conducted every three months for first six months after the operation, and thereafter every six months to look for anastomotic strictures.

## Results

A total of 13 patients were included for the present study. The diagnoses were, as follows: pCCA (*n* = 9), intrahepatic cholangiocarcinoma (*n* = 3), and gallbladder cancer (*n* = 1). The patient details are presented in Table 1. Eleven patients had jaundice before surgery, and two of these patients were treated with percutaneous transhepatic catheter drainage (PTCD) to relieve the jaundice before surgery. All patients underwent R0 resection. The mean operation time was 318.7 ± 54.0 min, and the mean estimated blood loss was 442.3 ± 288.1 ml. Tumor abutment to the hepatic artery or portal vein was present in seven patients, and the tumor could be completely removed without damaging the major vessels for all these patients. Segmental right hepatic artery invasion was detected in one patient. This patient underwent right hepatic artery resection and reconstruction. Furthermore, main portal vein invasion was detected in one patient. This patient underwent portal vein resection and reconstruction. No serious intraoperative events occurred. Three patients developed intra-abdominal collections. One of these patients had associated intraperitoneal infection, and all patients were successfully managed by ultrasound-assisted puncture and drainage. One patient suffered from postoperative ascites and bleeding, and recovered after conservative treatment. No perioperative death occurred, and none of the patients developed bile leakage or anastomotic stenosis. The details of the surgery are presented in Table 2.

**Table 1:** Patient demographics and characteristics

Variables		No./Median value
Gender	Male	4
	Female	9
Age (years)		61.9 ± 10.3
Preoperative concurrent diseases		
	Hypertension	3
	Diabetes	2
	Cardiovascular and pulmonary disease	1
	Cirrhosis	1
	Gout	1
Clinical diagnosis		
	Hilar cholangiocarcinoma	9
	Intrahepatic cholangiocarcinoma	3
	Gallbladder carcinoma	1
TNM staging	T1N0M0	1
	T2aN0M0	1
	T2bN1M0	1
	T3N0M0	2
	T3N1M0	3
	T4N0M0	1
	T4N1M0	4
Physical signs		
	Jaundice	11
	Abdominal pain	8
Bismuth-Corlette (only for hilar cholangiocarcinoma)		
	II	2
	IIIa	2
	IIIb	1
	IV	4
Previous history of surgery		2
Preoperative blood test		
	CA19-9 (U/L)	334.0 ± 455.0
	CEA (ng/L)	40.0 ± 286.2
	ALB (g/L)	34.2 ± 4.2
	Total bilirubin (µmol/L)	251.9 ± 173.8
	PT(s)	12.2 ± 2.2
Preoperative biliary drainage		
	PTCD	2
	None	11

**Table 2:** Patient demographics and characteristics

Variables		No./Median value
Operation time (minutes)		318.7 ± 54.0
Intraoperative blood loss (ml)		442.3 ± 288.1
Vascular invasion		
	Portal vein abutment	6
	Hepatic artery abutment	5
	Portal vein invasion	1
	Hepatic artery invasion	1
Type of hepatectomy		
	Ι+IVb	2
	Ι+IVb+V	3
	Ι+IV+V	1
	Ι+II+III+IV	2
	Ι+II+III+IV	1
	Perihilar resection	4
Number of bile duct stumps at the hilum after resection		
	3	2
	4	3
	5	6
	6	1
	8	1
Postoperative complications		
	Post-operative bile leakage	0
	Postoperative anastomotic stenosis	0
	Ascites	1
	Abdominal infection	1
	Intra-abdominal collections	3

The patients were followed up every 3 months after surgery when conditions permitted. We also observed the postoperative tumor recurrence. The time and number of recurrences were as follows: 6 months postoperatively (*n* = 2, 15.4%), 9 months postoperatively (*n* = 1, 7.7%), 12 months postoperatively (*n* = 1, 7.7%), 18 months postoperatively (*n* = 1, 7.7%), and no recurrence (*n* = 2, 15.4%). One of two recurrence-free patients had survived for more than 3 years and another for more than 5 years. Unfortunately, most of the patients were lost to follow-up after the discovery of tumor recurrence or 6 months after surgery, so complete survival data were not available.

## Discussion

The current study presented a modified technique of hepatojejunostomy for patients with perihilar tumors, which could reduce the incidence of postoperative anastomotic leak and stenosis. The first important step was to close the gap between the left and right branches of the portal vein, and the cut surface of the liver to enhance the integrity and firmness of the posterior wall of the anastomosis. Then, the posterior wall of the jejunum was sutured to the wall of the adjoining portal vein branches, the connective tissue, and the liver tissue below the lower edge of the bile duct to strengthen the anastomosis, since the wall of smaller bile ducts is often weak. Previous studies have also reported the use of the portal vein wall and connective tissue below the bile duct as a part of the posterior wall of the anastomosis, but the gap between the portal vein branches and liver section was not closed before anastomosis [10, 11, 13, 15, 16]. Subsequently, the biliary stumps, which were close to each other, especially the bile ducts used for the posterior wall of the anastomosis, were sutured together using absorbable sutures. With this step, the posterior wall of the bile ducts was aligned in a straight line as much as possible, and the difficulty in performing the anastomosis was reduced.

Most surgeons place silicone stent tubes in bile ducts, while some surgeons do not use any biliary stent [1, 16, 17]. Some surgeons place the distal end of the stents completely into the intestinal lumen, but others drain the bile out of the body. The disadvantage of an external drainage is that this causes bile loss, which in turn, can cause water and electrolyte imbalance, and the drainage tubes fixed to the abdominal wall are cumbersome to manage for patients. Internal drainage overcomes the disadvantages of external drainage, but the biliary drainage across the anastomosis cannot be confirmed in the postoperative period. In the present study, silicon or latex tubes were used for combined internal and the external biliary drainage as described in the Methods section. In this manner, the bile secretion can be observed after the operation, and the accumulation of fluid in the intestinal lumen near the anastomosis can be reduced. These would be helpful for the healing of the anastomosis, and reducing the bile loss, when compared to that of total external drainage.

In the present study, 4 − 0 prolene was used for the anastomosis, and the biliary stents were fixed to the jejunal wall using 4 − 0 vicryl. Vicryl sutures, which becomes completely absorbed within 60–90 days, can decrease the incidence of early postoperative bile duct strictures and stone formation [18, 19]. However, vicryl sutures are not smooth enough to slide through tissue, and are not preferred for continuous sutures, especially when the surgical field is difficult to expose. Prolene is very smooth and can easily pass through tissues helps to minimize the difficulty of the operation [20, 21]. Regrettably, prolene is not absorbable, and this may increase the risk of anastomotic stones [18]. Alternatively, polydioxanone (PDS) can be used for hepatojejunostomy in clinical practice [16, 17, 22]. The advantages and disadvantages of both absorbable and non-absorbable sutures were both taken into account in the present study.

The incidence of postoperative complications is an important factor in evaluating the success of a surgical technique. No serious complications, especially bile leakage and anastomotic stenosis, occurred in the study patients. Traditional cholangiojejunostomy is not only complicated to perform, but also time-consuming, and prone to biliary leakage, anastomotic stenosis, biliary fistula and reflux cholangitis after the operation [20]. Furthermore, the in-hospital mortality rates for conventional methods for pCCA is higher, when compared to other diseases that require liver resection [23].

Anastomotic leakage is caused by infection, ischemia, edema, faulty anastomotic techniques, and anastomotic tension [24–26]. In some conventional surgery for pCCAs, multiple thin biliary ducts (< 1–2 mm) are ligated, since the number of end-to-side cholangioenterostomy is technically restricted. The ligated bile ducts can dilate in the long term, and compress the portal vein within the Glissonian sheath, leading to portal hypertension and persistent cholestasis, and increasing the risk of cholangitis, bile leakage and septic events after the operation [1].

Anastomotic strictures are prone to occur when the anastomosis is very small, there is tension at the anastomotic site, and ischemia occurs due to aggressive dissection, fibrosis, and/or adhesions [25]. One of the ways to prevent strictures is to perform portoenterostomy for patients with complex perihilar bile duct strictures [11]. Some surgeons also perform portoenterostomy as a rescue procedure after major biliary complications following traditional cholangioenterostomy [27]. Kasai portoenterostomy can be used for patients with pCCA, since this is associated with a low incidence of postoperative bile duct stenosis. No anastomotic stenosis was detected in the present study up to six months of follow up after the operation.

The modified hepatojejunostomy also provides an opportunity for resection in pCCA patients with high preoperative jaundice. For example, in some patients with HCCA type IIIa and IIIb, right hepatectomy/ trisectionectomy or left hepatectomy may not be possible due to various reasons such as high preoperative bilirubin or poor compliance and can’t wait for biliary drainage before surgery. In such cases, local excision can be performed with reconstruction using modified technique. Hence, except for two patients, none of the patients in this study underwent preoperative biliary drainage in this study.

The present study had some limitations. First, the present study was a single-center retrospective study with a small sample size. This is because only selected patients with various special conditions were included in this study. Second, the short-term complication profile is favorable, but that confirmation of anastomotic durability and late complication rates still needs a larger series with longer follow-up. Third, there was a lack of sufficient follow-up data due to various reasons, and the impact of the described technique on overall survival was not determined.

## Conclusion

In conclusion, the investigators recommend the modified hepatojejunostomy technique as a safe and effective technique for bilioenteric reconstruction after the resection of tumors affecting the perihilar region, with the presence of multiple bile duct stumps for reconstruction. The recommended technique is easy to learn and simpler to perform for difficult cases. Future long-term multi-center studies are required to validate the findings of the present study.

## Data Availability

All data generated or analyzed during this study are included in this published article.

## References

[1] Aydin U, Yedibela S, Yazici P, et al. A new technique of biliary reconstruction after high hilar resection of hilar cholangiocarcinoma with tumor extension to secondary and tertiary biliary radicals. Ann Surg Oncol. 2008;15:1871–9. 10.1245/s10434-008-9926-x. 18454297 10.1245/s10434-008-9926-x

[2] Jarnagin WR, Fong Y, DeMatteo RP, et al. Staging, resectability, and outcome in 225 patients with hilar cholangiocarcinoma. Ann Surg. 2001;234:507–17. 10.1097/00000658-200110000-00010. 11573044 10.1097/00000658-200110000-00010 PMC1422074

[3] Silva MA, Tekin K, Aytekin F, et al. Surgery for hilar cholangiocarcinoma; a 10 year experience of a tertiary referral centre in the UK. Eur J Surg Oncol. 2005;31:533–9. 10.1016/j.ejso.2005.02.021. 15922889 10.1016/j.ejso.2005.02.021

[4] Jarnagin WR, Bowne W, Klimstra DS, et al. Papillary phenotype confers improved survival after resection of hilar cholangiocarcinoma. Ann Surg. 2005;241:703–12. 10.1097/01.sla.0000160817.94472.fd. 15849506 10.1097/01.sla.0000160817.94472.fd PMC1357125

[5] Cannon RM, Brock G, Buell JF. Surgical resection for hilar cholangiocarcinoma: experience improves resectability. HPB. 2012;14:142–9. 10.1111/j.1477-2574.2011.00419.x. 22221577 10.1111/j.1477-2574.2011.00419.x PMC3277058

[6] Xiang S, Lau WY, Chen XP. Hilar cholangiocarcinoma: controversies on the extent of surgical resection aiming at cure. Int J Colorecta l Dis. 2015;30:159–71. 10.1007/s00384-014-2063-z. 10.1007/s00384-014-2063-z PMC4304009 25376337

[7] Li QJ, Zhou ZG, Lin XJ, et al. Clinical practice of basin-shaped hepaticojejunostomy following hilar resection of stage III/IV hilar cholangiocarcinoma. BMC Gastroenterol. 2019;19:99. 10.1186/s12876-019-1012-2. 31221103 10.1186/s12876-019-1012-2 PMC6585136

[8] Yang XJ, Dong XH, Chen SY, et al. Application of multiple roux-en-Y hepaticojejunostomy reconstruction by formation of bile hilar duct lake in the operation of hilar cholangiocarcinoma. World J Clin Cases. 2020;8:68–75. 10.12998/wjcc.v8.i1.68. 31970171 10.12998/wjcc.v8.i1.68 PMC6962072

[9] Ha TY, Hwang S, Song GW, et al. Cluster hepaticojejunostomy is a useful technique enabling Secure Reconstruction of severely damaged hilar bile ducts. J Gastrointest Surg. 2015;19:1537–41. 10.1007/s11605-015-2844-x. 25956723 10.1007/s11605-015-2844-x

[10] Hwang S, Ha TY, Song GW, Jung DH. Cluster hepaticojejunostomy with radial spreading anchoring traction technique for secure reconstruction of widely opened hilar bile ducts. Korean J Hepatobiliary Pancreat Surg. 2016;20:66–70. 10.14701/kjhbps.2016.20.2.66. 27212993 10.14701/kjhbps.2016.20.2.66 PMC4874047

[11] Kasai MKS, Asakura Y, Suzuki H, et al. Surgical treatment of billary atresia. J Pediatr Surg. 1968;3:665–75.

[12] Gao JB, Bai LS, Hu ZJ, et al. Role of Kasai procedure in surgery of hilar bile duct strictures. World J Gastroenterol. 2011;17:4231–4. 10.3748/wjg.v17.i37.4231. 22072856 10.3748/wjg.v17.i37.4231 PMC3208369

[13] Mimmo A, Tzedakis S, Gueroult P, et al. Kasai-Like Portoenterostomy for multiple biliary Duct Reconstruction after Extended Liver Resection of Perihilar Cholangiocarcinoma. Ann Surg Oncol. 2021;28:7741. 10.1245/s10434-020-09551-x. 33993375 10.1245/s10434-020-09551-x

[14] Chen XP, Huang ZY, Chen YF, et al. Improvement of biliary Reconstruction after Resection of Hilarcholangiocarcinoma. Zhonghua Waike Zazhi. 2008;46:634–5. 10.3321/j.issn:0529-5815.2008.08.024. http://dx.chinadoi.cn/.

[15] Nimura Y. Radical surgery of left-sided klatskin tumors. HPB. 2008;10:168–70. 10.1080/13651820801992674. 18773047 10.1080/13651820801992674 PMC2504368

[16] Dilek ON, Gungor F, Acar T, et al. The role of Portoenterostomy with aggressive Hilar dissection in biliary tract tumors: report of Case Series and Review of the literature. Indian J Surg. 2021;83:114–20. 10.1007/s12262-020-02259-y. 32410790 10.1007/s12262-020-02259-y PMC7222060

[17] Bednarsch J, Czigany Z, Heise D, et al. Leakage and stenosis of the Hepaticojejunostomy following surgery for Perihilar Cholangiocarcinoma. J Clin Med. 2020;9:1392. 10.3390/jcm9051392. 32397289 10.3390/jcm9051392 PMC7290596

[18] Li Q, Tao L, Wu X, Mou L, et al. Bile duct stone formation around a Prolene suture after cholangioenterostomy. Pak J Med Sci. 2016;32:263–6. 10.12669/pjms.321.8985. 27022388 10.12669/pjms.321.8985 PMC4795881

[19] Javed AA, Mirza MB, Sham JG, et al. Postoperative biliary anastomotic strictures after pancreaticoduodenectomy. HPB. 2021;23:1716–21. 10.1016/j.hpb.2021.04.008. 34016543 10.1016/j.hpb.2021.04.008

[20] Lu BC, Ren PT. Treatment of hilar cholangiocarcinoma of Bismuth-Corlette type III with hepaticojejunostomy. Contemp Oncol (Pozn). 2013;17:298–301. 10.5114/wo.2013.35274. 24596518 10.5114/wo.2013.35274 PMC3934065

[21] Yang LY, Luo Q, Lu L, et al. Increased neutrophil extracellular traps promote metastasis potential of hepatocellular carcinoma via provoking tumorous inflammatory response. J Hematol Oncol. 2020;13:3. 10.1186/s13045-019-0836-0. 31907001 10.1186/s13045-019-0836-0 PMC6945602

[22] Naseer F, Lin CH, Lin TS et al. Long-term Results in Comparative Analysis of Merits in Using Polypropylene and Polydioxanone for Microsurgical Biliary Reconstruction in Living Donor Liver Transplantation. *Transplant. Proc* 2020; 52: 233–8. 10.1016/j.transproceed.2019.11.009. 10.1016/j.transproceed.2019.11.009 31870604

[23] Sakata J, Shirai Y, Tsuchiya Y et al. Preoperative cholangitis independently increases in-hospital mortality after combined major hepatic and bile duct resection for hilar cholangiocarcinoma. Langenbecks. Arch. Surg. 2009; 394: 1065–72. 10.1007/s00423-009-0464-1 10.1007/s00423-009-0464-1 19169703

[24] Brunner M, Stockheim J, Krautz C et al. Continuous or interrupted suture technique for hepaticojejunostomy? A national survey. BMC Surg. 2018; 18:84. 10.1186/s12893-018-0418-z 30309351 10.1186/s12893-018-0418-z PMC6182832

[25] Antolovic D, Koch M, Galindo L, et al. Hepaticojejunostomy–analysis of risk factors for postoperative bile leaks and surgical complications. J Gastrointest Surg. 2007;11:555–61. 10.1007/s11605-007-0166-3. 17394045 10.1007/s11605-007-0166-3

[26] Hirano S, Tanaka E, Tsuchikawa T, et al. Techniques of biliary reconstruction following bile duct resection (with video). J Hepatobiliary Pancreat Sci. 2012;19:203–9. 10.1007/s00534-011-0475-5. 22081253 10.1007/s00534-011-0475-5 PMC3311849

[27] Sharma A, Hammond JS, Psaltis E, et al. Portoenterostomy as a Salvage Procedure for major biliary complications following Hepaticojejunostomy. J Gastrointest Surg. 2017;21:1086–92. 10.1007/s11605-017-3372-7. 28181137 10.1007/s11605-017-3372-7

